# Research on Quantum-Attack-Resistant Strong Forward-Secure Signature Schemes

**DOI:** 10.3390/e25081159

**Published:** 2023-08-02

**Authors:** Fengyin Li, Junhui Wang, Mengxue Shang, Dandan Zhang, Tao Li

**Affiliations:** School of Computer Science, Qufu Normal University, Rizhao 276800, China; wangjunhui0529@163.com (J.W.); shangmx1214@163.com (M.S.); zdd202302@163.com (D.Z.); litao_2019@qfnu.edu.cn (T.L.)

**Keywords:** lattice, quantum-attack-resistant, key-iteration algorithm, strong forward-secure signature, remote user authentication

## Abstract

The security of digital signatures depends significantly on the signature key. Therefore, to reduce the impact of leaked keys upon existing signatures and subsequent ones, a digital signature scheme with strong forward security could be an effective solution. Most existing strong forward-secure digital signature schemes rely on traditional cryptosystems, which cannot effectively resist quantum attacks. By introducing lattice-based delegation technology into the key-iteration process, a two-direction and lattice-based key-iteration algorithm with strong forward security is proposed. In the proposed algorithm, a unique key pair is assigned to the signer in every period. Based on the proposed algorithm, a strong forward-secure signature scheme is further put forward, which achieves resistance to quantum attacks. Performance analysis shows that under the security assumption of the SIS problem on the lattice, the proposed strong forward-secure signature scheme is existentially unforgeable under the random oracle model. Ultimately, based on the proposed strong forward-secure signature scheme, a remote identity-authentication scheme that is resistant to quantum attacks is proposed, ensuring post-quantum security in the user-authentication process.

## 1. Introduction

As one of the essential tools of digital authentication, digital signatures are widely applied to e-commerce and network communication. The security of digital signatures depends largely on the signature key. Leaked keys threaten entire signature systems, and entire systems can collapse due to key leakage. Leaked private keys render all signatures generated by them untrustworthy. Therefore, to ensure the legitimacy of the signature, the signer must invalidate previous signatures and rebuild a new signature system before signing.

To address the above problems, Anderson proposed the concept of forward security at the ACM CSS conference in 1997 [1]. They achieved forward security by updating the keys. In 2000, Anderson further produced the concept of backward security [2]. Backward security ensures that the leakage of the current key will not hamper future signing. In 2001, Burmester et al. put forward the concept of strong forward security [3], which further improves the security of the signature system. A strong forward-secure signature scheme can ensure both forward security and backward security.

Since then, the strong forward-secure signature scheme has been deeply studied. Cheng Yage et al. proposed a dynamic threshold signature scheme with strong forward security [4]. Li Fengyin et al. put forward a privacy-aware PKI model with strong forward security [5]. Yoneyama put forward a one-round authenticated key exchange with strong forward secrecy in the standard model against a constrained adversary [6]. The above signature schemes are, respectively based on the Chinese remainder theorem, RSA, and the Diffie–Hellman difficult problem, which cannot resist quantum attacks.

Identity-based Cryptography (IBC) has received great attention due to its efficiency in key management [7]. To ensure the security of signatures in the case of key leakage, the identity-based strong forward-security signature scheme was studied. However, these identity-based strong forward-security signature schemes are based on traditional cryptosystems, which cannot resist quantum attacks.

Unlike classical solutions, quantum digital signatures use quantum laws to sign a document with information-theoretical integrity, authenticity, and non-repudiation [8]. Quantum laws refer to the laws of quantum mechanics. Quantum cryptography applies the basic principles of quantum mechanics, such as the uncertainty principle, quantum no-cloning theorem, and quantum entanglement characteristics, to ensure the security of quantum cryptography [9]. Gottesman and Chung pioneered a quantum digital signature scheme based on the basic principles of quantum physics in 2001 [10]. The research of quantum digital signatures is still in an active stage, so there is no widely used standardization scheme. Various things can be called quantum digital signatures [11].

Quantum key distribution also exploits the properties of quantum mechanics to secure communications. It enables both parties in communication to generate and share a random, secure key. Quantum key distribution is only used to generate and distribute keys, and does not transmit any real messages. The current blockchain platform relies on digital signatures and is vulnerable to attacks by a quantum computer. Kiktenko and Pozhar proposed to introduce quantum key distribution into the blockchain [12]. Due to the tremendous progress in the deployment of quantum key distribution, practical secure quantum key distribution protocols are also being investigated [13]. The implementation of quantum key distribution involves highly specialized technologies and equipment, which increases the cost of implementation and maintenance. Realizing a perfect quantum key distribution system is a lengthy process due to challenges such as technical limitations and infrastructure requirements in practical applications [14].

From the perspective of practicality, post-quantum cryptography has higher practicability at present. Consistent with the goal of quantum cryptography, the research of post-quantum cryptography is also to protect communication and data security from attacks by a quantum computer. There are four mainstream post-quantum cryptography algorithms: lattice-based, encode-based, hash-based, and multivariate-based [15]. Lattice-based algorithms are considered to be one of the most promising post-quantum encryption algorithms because of their better balance among security, public-key size, private key size, and computation speed [16]. Post-quantum cryptography technologies are being explored to develop cryptography algorithms that remain secure in the presence of quantum adversaries. Although these algorithms show promising prospects, more research is needed to ensure their security, efficiency, and widespread application in the face of quantum threats [17].

Therefore, the lattice-based signature scheme with quantum-resistant attacks has become a research hotspot. Kansals et al. proposed group signature from lattices preserving forward security in a dynamic setting [18]. Liao et al. put forward a fully dynamic forward-secure group signature from lattice [19]. Le et al. put forward lattice blind signatures with forward security [20]. Wu et al. presented an efficient identity-based forward-secure signature scheme from lattices [21]. Zhang et al. raised a lattice-based strongly unforgeable forward-secure identity-based signature scheme with a flexible key update [22]. All the above signature schemes neglect backward security. To solve this problem, we propose a novel key-iteration algorithm, upon which a signature scheme is further proposed, to achieve strong forward security. The proposed signature scheme could guarantee quantum-attack-resistant strong forward security.

## 2. Preliminaries

### 2.1. Framework of Strong Forward-Secure Signature Scheme

A strong forward-secure signature scheme consists of the following four polynomial time algorithms.

**Figure 1:**
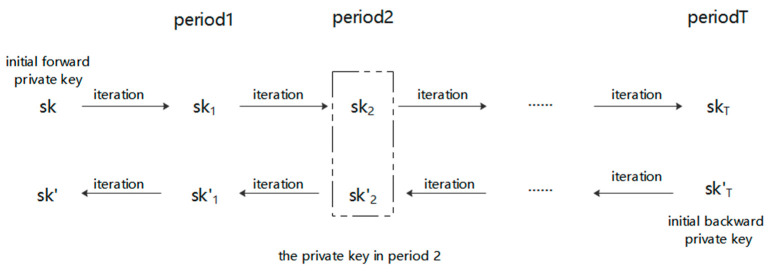
The private key-iteration process of the strong forward-secure signature scheme.

1. Parameter generation: input security parameter n, output public parameter PP, master key msk and user initial key usk.
2. Key iteration and update: When the user $U_{k}$ wants to use the private key, he initiates a key request to PKG and sends the identity ${ID}_{U_{k}}$ together. PKG inputs the public parameters PP, master key msk, user initial key usk and user identity ${ID}_{U_{k}}$, then executes the algorithm to generate the initial forward private key ${sk}_{{ID}_{U_{k}}||0}$ and initial backward private key ${sk’}_{{ID}_{U_{k}}||T}$. When iterating the forward private key, user inputs the current period i, user identity ${ID}_{U_{k}}$ and current forward private key ${sk}_{{ID}_{U_{k}}||i}$, then outputs the forward private key ${sk}_{{ID}_{U_{k}}||i+1}$ for the next period i + 1. When iterating the backward private key, user inputs the current period i, user identity ${ID}_{U_{k}}$ and current backward private key are input ${sk’}_{{ID}_{U_{k}}||i}$, then outputs the backward private key ${sk}_{{ID}_{U_{k}}||i-1}$ for the previous period i − 1. The private key of the i-th period is ${SK}_{{ID}_{U_{k}}||i}$ = ${sk}_{{ID}_{U_{k}}||i}+{sk’}_{{ID}_{U_{k}}||i}$, which is the result of concatenating the forward private key and the backward private key. The iteration process of the private key is shown in Figure 1.
3. Signature generation: User inputs his identity ${ID}_{U_{k}}$, the private key ${SK}_{{ID}_{U_{k}}||i}$ of the current period i and the message m, then outputs the signature $e_{i}$ at this period.
4. Signature verification: The verifier inputs the user’s identity ${ID}_{U_{k}}$, the public key ${PK}_{{ID}_{U_{k}}||i}$ of the current period i, the original message m and the signature $e_{i}$, if the signature is valid then accept it, otherwise reject it.

### 2.2. Security Model

The identity-based strong forward-secure signature scheme is existentially unforgeable under adaptive chosen-message attack. The security of the model is defined using a game in which challenger C and adversary A interact. 

Parameter establishment: The challenger runs the parameter generation algorithm and sends the generated public parameter PP to the adversary while keeping the master key msk and user master key usk for itself.

Queries: Adversary A adaptively issues many different following queries to the challenger:

1. Key query: A own the ability to ask any identity ${ID}_{U_{k}}$(k = 1,2,…,N) for the key of any period i (i ≤ T), and C generates the key ${SK}_{{ID}_{U_{k}}||i}$ of identity ${ID}_{U_{k}}$ in period i and sends it to A.
2. Signature query: A can inquire about the signature of any identity ${ID}_{U_{k}}$ in any period i (i ≤ T), and C generates the signature $e_{i}$ of the identity ${ID}_{U_{k}}$ in period i and sends it to A.

Forgery: A outputs an identity ${ID}_{U_{k}}^{*}$, period $i^{*}$, message $m^{*}$ and signature ${e_{i}}^{*}$. If the ${ID}_{U_{k}}^{*}$ has not been subjected to key inquiry and signature inquiry, and the signature ${e_{i}}^{*}$ will be verified to pass, then A wins the game. The advantage of A winning is:

$${Adv}_{A,C}^{Unforge}(\lambda ):=Pr[A\;wins]=negl(\lambda )$$

### 2.3. Lattices and Hardness Assumptions

**Definition** **1** ([23] Lattice)**.** Lattice is a collection of linear combinations of all integer coefficients of n linearly independent vector groups $⋀=L(x_{1},x_{2},\cdots \cdots x_{n})=\left\{\sum_{i=1}^{n}a_{i}b_{i}|a_{i}\in Z\right\}$, namely: $x_{1},x_{2},\cdots \cdots x_{n}$.

**Definition** **2** ([24] Full-rank lattice)**.** Define the m-dimensional full-rank q-ary lattice as: ${⋀}_{q}^{⊥}(A)=\left\{x\in Z^{m}|Ax=0(mod\;q)\right\}$, ${⋀}_{q}^{u}(A)=\left\{x\in Z^{m}|Ax=u(mod\;q)\right\}$. Among them, q is a prime number, m and n are positive integers, matrix $A\in Z_{q}^{n\times m}$, and vector $u\in Z_{q}^{n}$. ${⋀}_{q}^{⊥}(A)$ and ${⋀}_{q}^{u}(A)$ can be abbreviated as ${⋀}^{⊥}(A)$ and ${⋀}^{u}(A)$.

**Definition** **3** ([24] SIS problem)**.** Given an integer q, a matrix $A\in Z_{q}^{n\times m}$ and a real number β, find a non-zero vector e such that Ae = 0 mod q 0<||e||≤β and such a problem is called an SIS problem. The SIS problem is considered to be a difficult computational problem, where a solution that satisfies the conditions cannot be found within the effective time. Based on this difficult assumption, the SIS problem is widely used to construct lattice-based Cryptography schemes.

**Definition** **4** ([24] Gaussian distribution)**.** For any positive parameter σ ∈ R and any vector a ∈ $R^{n}$, there is $\rho_{\sigma ,a}(x)=exp\left(-\pi \frac{{\left||x-a|\right|}^{2}}{\sigma^{2}}\right)$.

**Definition** **5** ([24,25] Trapdoor-Generation Algorithm)**.** There is a PPT algorithm, given a prime number q ≥ 3, positive integer m ≥ 6 nlogq, security parameter n, run algorithm TrapGen(q,n) → (A,T), output a set of bases $T\in Z^{m\times m}$ of matrix $A\in Z_{q}^{n\times m}$ and lattice ${⋀}^{⊥}\;(A)$, so that the distribution of A and the uniform distribution on $Z_{q}^{n\times m}$ are statistically Indistinguishable, and the conditions ||T|| ≤ O(nlbq) and ||₸|| $\leq O(\sqrt{nlbq})$ hold. where ₸ represents the basis after Gram-Schmidt orthogonalization of T. Trapdoor is a special type of key, usually generated in a public-key cryptosystem, which can achieve specific security functions such as encryption, signature, identity authentication, etc.

**Definition** **6** ([26] Lattice-basis delegation algorithm)**.** Let A∈$Z_{q}^{n\times m}$ be a full-rank matrix, matrix $R\in D_{m\times m}$, T is a set of bases of lattice ${⋀}^{⊥}(A)$, Gaussian parameters satisfy $\sigma >||₸||\cdot \sigma_{R}\sqrt{m}\cdot \omega ({lb}^{3/2}m)$. The Gaussian parameter $\sigma_{R}$ satisfies $\sigma_{R}=\sqrt{nlbq}\cdot \omega (\sqrt{lbm})$, $D_{m\times m}$ represents the matrix distribution in $Z^{m\times m}$ that satisfies ${\left(D_{\sigma_{R}}^{m}\right)}^{m}$ and the modulo q is invertible. Then there is a PPT algorithm BasisDel(A,R,T,σ) that can output a set of bases $T_{B}$ for the lattice ${⋀}^{⊥}\;(AR^{-1})$, such that $\left||T_{B}||<\sigma /\omega (\sqrt{lbmq}\right)$. The generation of a set of lattice trapdoors is a relatively complex process. In some cases, when multiple pairs of lattice trapdoors are required, the lattice-basis delegation algorithm can be utilized to quickly generate another pair of related new lattice bases from a known pair.

**Definition** **7** ([24] Difficulty specification of small integer solution problems)**.** Knowing any polynomial bounded real number m,β=poly(n) and prime numbers $q\geq \beta \cdot \omega (\sqrt{nlbn})$, the difficulty of solving the SIS problem with average instances is comparable to that of solving the approximate shortest independent vectors problem with the worst-case on the lattice (shortest independent vectors problem, ${SIVP}_{\gamma }$), where $\gamma =\beta \cdot O(\sqrt{n})$.

**Definition** **8** (Hash function)**.** Randomly select prime numbers q, n, m>64+nlogn/log3, and define the following hash functions:$H_{1}:{\left\{0,1\right\}}^{*}\to Z^{m\times m}$,$H_{2}:{\left\{0,1\right\}}^{*}\to \left\{v:v\in {\left\{-1,0,1\right\}}^{k},||v||\leq k\right\}$.

**Lemma** **1** ([27] Rejection sampling)**.** Let V be a subset of above $Z^{m}$, and the norm of the elements of V does not exceed T, r ∈ R exists, $r=\omega (T\sqrt{logm})$, h:V → R is a probability distribution, there is a constant M = O(1)) such that The probability of the distribution satisfying the following two algorithms is statistically asymptotic:

v ← h, z ← $D_{v,r}^{m}$, output signature(v,z) with probability $min(1,\frac{D_{r_{i}}^{m}(z)}{{MD}_{v,r}^{m}(z)})$;
v ← h, z ← $D_{r}^{m}$, output the signature (v,z) with probability $\frac{1}{M}$.

**Lemma** **2** ([28] Fork Lemma)**.** Let q be a positive integer and H be a set with h > 2 elements. Let IG be a parameter generation algorithm, B is a random algorithm, the input of algorithm B is {x,$h_{1}$,…,$h_{q}$}, and the output is (J,σ), where x∈{0,…,q}, $h_{i}$∈H (i∈[q]). Let the acceptance probability acc of algorithm B be the probability that J ≥ 1 in the trial EXP = [ x ← IG; $h_{1}$,…,$h_{q}$←H; (J,σ) ← B(x,$h_{1}$,…,$h_{q}$)].Let the fork algorithm${\;F}_{B}\;$related to B is expressed as follows:

1. Algorithm $F_{B}$ input x;
2. Randomly select ρ ∈ {0,1};
3. Randomly select $h_{1}$,…,$h_{q}$ from the set H;
4. (I,σ) ← B(x,$h_{1}$,…,$h_{q}$; ρ);
5. If I = 0, return (0, ε, ε);
6. Randomly select ${h’}_{1}$,…,${h’}_{q}$ from the set H;
7. (I’, σ’) ← B(x, $h_{1}$, … , $h_{I-1}$, ${h’}_{I}$, … , ${h’}_{q}$; ρ);
8. If I = I ’ and h≠h’, output (1, σ, σ’’); otherwise output (0, ε, ε), let frk=Pr[b=1,x←IG; (b,σ,σ’)←$F_{B}(x)$], then frk≥acc$\cdot (\frac{acc}{q}-\frac{1}{h})$.

The random oracle model (ROM) is a universal model for proving the security of digital signature schemes. Under the ROM model, an important technology to prove the security of the scheme is the random oracle replay technology, i.e., to solve a hard problem of consciousness by replaying the hash value. The theoretical basis of this technique is the Fork Lemma.

## 3. A Strong Forward-Secure Signature Scheme Based on Identity on Lattice

To achieve quantum-attack-resistant security, this section introduces a lattice-basis delegation technology into the key-iteration process and proposes a key-iteration algorithm. This algorithm divides the key into T periods and assigns a unique key pair to each period through forward and backward iterations of two initial keys, which ensures strong forward security of the key. Then, an identity-based signature scheme with strong forward security that can resist quantum attacks is constructed using the proposed key-iteration algorithm.

### 3.1. Strong Forward-Security Key-Iteration Algorithm

Generating a set of lattice bases is relatively complex using the trapdoor-generation algorithm. However, when multiple pairs of lattice bases are needed, the lattice-delegation technology can quickly generate another pair of related new lattice bases based on a known pair. To ensure the security of signatures after the private key is leaked, this section introduces lattice-delegation technology into the key-iteration process, and proposes a bidirectional key-iteration algorithm with strong forward security. The proposed algorithm assigns a unique key pair for each period, therefore ensuring the forward security and backward security of the key. Specifically, in the key-iteration process, PKG divides the key into T periods, the signatures of different periods are relatively independent, and it is impossible to generate keys of other periods from the keys of a certain period. This solves the problem of whether the signature is still legal after the private key is leaked. The key-iteration algorithm for strong forward security is as follows:

#### 3.1.1. Symbol Description

The specific meanings of the symbols used in the strong forward-security signature scheme constructed in this paper are shown in Table 1.

#### 3.1.2. System Initialization

The strong forward-secure key-iteration algorithm has two entities: PKG and user. First, PKG performs parameter generation and master key generation, then publishes the parameters and sends the master key to the user through a secure channel. Users use the master key and the user key for key iteration and update.

1. System parameter generation

PKG generates parameters, Setup(n) → PP:PKG inputs security parameter n, then randomly selects a prime number q, the prime m>64+nlogn/log3, a Gaussian parameter $\sigma_{R}$ satisfies the relation $\sigma_{R}=\sqrt{nlbq}\cdot \omega (\sqrt{lbm})$, and a hash function $H_{1}$. Then PKG publishes the parameters $PP=(n,q,m,\sigma_{R}{,H}_{1})$.

2. Master key generation

PKG generates master key, KeyGen(PP) → $\left\{(M_{U_{k}A_{0}},M_{U_{k}T_{A_{0}}}),(M_{U_{k}B_{0}},M_{U_{k}T_{B_{0}}})\right\}$: PKG inputs the public parameter PP, and generates the master key through the trapdoor-generation algorithm.

$TrapGen(q,n)\to (M_{U_{k}A_{0}},M_{U_{k}T_{A_{0}}})$, $TrapGen(q,n)\to (M_{U_{k}B_{0}},M_{U_{k}T_{B_{0}}})$, where $M_{U_{k}T_{A_{0}}}$ and $M_{U_{k}T_{B_{0}}}$ are the user’s master private key i.e., $msk=(M_{U_{k}T_{A_{0}}},M_{U_{k}T_{B_{0}}})$,$M_{U_{k}A_{0}}$ and $M_{U_{k}B_{0}}$ are the user’s master public key i.e., $mpk=(M_{U_{k}A_{0}},M_{U_{k}B_{0}})$. PKG transmits msk and mpk to users through a secure channel.

3. User master key generation

User $U_{k}$(k = 1,2,…,N) generates user master key using public parameter PP. $KeyGen(PP)\to (U_{kA_{0}},U_{kT_{A_{0}}}),(U_{kB_{0}},U_{kT_{B_{0}}})\}$: The user $U_{k}$ inputs the public parameter PP, and generates the user master key through the trapdoor-generation algorithm. $TrapGen(q,n)\to (U_{kA_{0}},U_{kT_{A_{0}}})$, $TrapGen(q,n)\to (U_{kB_{0}},U_{kT_{B_{0}}})$, where $U_{kT_{A_{0}}}$ and $U_{kT_{B_{0}}}$ are the user master private key i.e., $usk=(U_{kT_{A_{0}}},U_{kT_{B_{0}}})$, $U_{kA_{0}}$ and $U_{kB_{0}}$ are the user master public key i.e., $upk=(U_{kA_{0}},U_{kB_{0}})$. In addition, the user $U_{k}$ selects two sets of Gaussian parameters $\sigma =(\sigma_{0},\sigma_{1},\cdots \cdots ,\sigma_{T})$, $\sigma ’=({\sigma ’}_{0},{\sigma ’}_{1},\cdots \cdots ,{\sigma ’}_{T})$, to satisfies $\sigma >||{Ū}_{kT_{A_{0}}}||\cdot \sigma_{R}\sqrt{m}\cdot \omega ({lb}^{3/2}m)$ and σ’> $\left||{Ū}_{kT_{B_{0}}}||\cdot \sigma_{R}\sqrt{m}\cdot \omega ({lb}^{3/2}m\right)$. Where ${Ū}_{kT_{A_{0}}}$ and ${Ū}_{kT_{B_{0}}}$ are, respectively, the basis of $U_{kT_{A_{0}}}$, $U_{kT_{B_{0}}}$ after Gram-Schmidt orthogonalization.

#### 3.1.3. Key-Iteration Algorithm

User $U_{k}$ performs key iteration using identity and master public and private key pair. Iteration $\left(M_{U_{k}T_{A_{0}}},M_{U_{k}T_{B_{0}}},{M_{U_{k}A_{0}},M_{U_{k}B_{0}},U}_{kT_{A_{0}}},U_{kT_{B_{0}}},{ID}_{U_{k}}\right)$ → (${SK}_{U_{k}}$, ${PK}_{U_{k}}$): The user $U_{k}$ enters the master private key $\left(M_{U_{k}T_{A_{0}}},M_{U_{k}T_{B_{0}}}\right)$, the master public key ${(M}_{U_{k}A_{0}},M_{U_{k}B_{0}})$, the user master private key $\left(U_{kT_{A_{0}}},U_{kT_{B_{0}}}\right)$ and the identity ${ID}_{U_{k}}$ of the user $U_{k}$. During the key-iteration process, the user performs the following operations:

1. Forward private key iterative algorithm

The user $U_{k}$ generates the initial forward private key at period t = 0: $R_{{ID}_{U_{k}}||0}=H_{1}({ID}_{U_{k}}||U_{kT_{A_{0}}}||0)$, $A_{{ID}_{U_{k}}||0}=M_{U_{k}A_{0}}\cdot {\left(R_{{ID}_{U_{k}}||0}\right)}^{-1}$, $BasisDel(M_{U_{k}A_{0}},R_{{ID}_{U_{k}}||0},M_{U_{k}T_{A_{0}}},\sigma_{0})\to {sk}_{{ID}_{U_{k}}||0}$, where ${sk}_{{ID}_{U_{k}}||0}$ is the initial forward private key with forward security;

The user $U_{k}$ iterates the forward private key from period i − 1 to period i: $R_{{ID}_{U_{k}}||i-1}=H_{1}({ID}_{U_{k}}||U_{kT_{A_{0}}}||i-1)H_{1}({ID}_{U_{k}}||U_{kT_{A_{0}}}||i-2)\cdots \cdots H_{1}({ID}_{U_{k}}||U_{kT_{A_{0}}}||1)H_{1}({ID}_{U_{k}}||U_{kT_{A_{0}}}||0)$, $A_{{ID}_{U_{k}}||i-1}=M_{U_{k}A_{0}}\cdot {\left(R_{{ID}_{U_{k}}||i-1}\right)}^{-1}$, and compute the $R_{i}=H_{1}({ID}_{U_{k}}||U_{kT_{A_{0}}}||i)$, then use algorithm$BasisDel(A_{{ID}_{U_{k}}||i-1},R_{i},{sk}_{{ID}_{U_{k}}||i-1},\sigma_{i})\to {sk}_{{ID}_{U_{k}}||i}$, since the forward private key ${sk}_{{ID}_{U_{k}}||i}$ of the i-th period is generated by the forward private key ${sk}_{{ID}_{U_{k}}||i-1}$ of the i − 1 period through the hash function and lattice-basis delegation algorithm, which ensures that the forward private keys ${({sk}_{{ID}_{U_{k}}||0},\cdots \cdots ,sk}_{{ID}_{U_{k}}||i-1},{sk}_{{ID}_{U_{k}}||i},\cdots \cdots ,{sk}_{{ID}_{U_{k}}||T})$ have forward-secure.

2. Backward private key-iteration algorithm

The user $U_{k}$ generates the initial backward private key in the period t=T: ${R’}_{{ID}_{U_{k}}||T}=H_{1}({ID}_{U_{k}}||U_{kT_{B_{0}}}||T)$, and compute the ${A’}_{{ID}_{U_{k}}||T}=M_{U_{k}B_{0}}\cdot {\left({R’}_{{ID}_{U_{k}}||T}\right)}^{-1}$, then use algorithm $BasisDel(M_{U_{k}B_{0}},{R’}_{{ID}_{U_{k}}||T},M_{U_{k}T_{B_{0}}},{\sigma ’}_{T})\to {sk’}_{{ID}_{U_{k}}||T}$, where ${sk’}_{{ID}_{U_{k}}||T}$ is the initial backward private key with backward security.

The user $U_{k}$ iterates the backward private key from period i to period i − 1: ${R’}_{{ID}_{U_{k}}||i}=H_{1}({ID}_{U_{k}}||U_{kT_{B_{0}}}||T)H_{1}({ID}_{U_{k}}||U_{kT_{B_{0}}}||T-1)\cdots \cdots H_{1}({ID}_{U_{k}}||U_{kT_{B_{0}}}||i+1)H_{1}({ID}_{U_{k}}||U_{kT_{B_{0}}}||i)$, ${A’}_{{ID}_{U_{k}}||i}=M_{U_{k}B_{0}}\cdot {\left({R’}_{{ID}_{U_{k}}||i}\right)}^{-1}$, and compute the ${R’}_{i-1}=H_{1}({ID}_{U_{k}}||U_{kT_{B_{0}}}||i)$, then use algorithm $BasisDel({A’}_{{ID}_{U_{k}}||i},{R’}_{i-1},{sk’}_{{ID}_{U_{k}}||i},{\sigma ’}_{i-1})\to {sk’}_{{ID}_{U_{k}}||i-1}$, since the backward private key ${sk’}_{{ID}_{U_{k}}||i-1}$ of the i − 1th period is generated by the backward private key ${sk’}_{{ID}_{U_{k}}||i}$ of the i-th period through the hash function and lattice-basis delegation algorithm, which ensures the backward private keys ${({sk’}_{{ID}_{U_{k}}||0},\cdots \cdots ,sk’}_{{ID}_{U_{k}}||i-1},{sk’}_{{ID}_{U_{k}}||i},\cdots \cdots ,{sk’}_{{ID}_{U_{k}}||T})$ have backward secure.

The private key of the user $U_{k}$ in period i is ${SK}_{{ID}_{U_{k}}||i}={sk}_{{ID}_{U_{k}}||i}+{sk’}_{{ID}_{U_{k}}||i}$. ${SK}_{U_{k}}=({SK}_{{ID}_{U_{k}}||0},{SK}_{{ID}_{U_{k}}||1},\cdots \cdots {,SK}_{{ID}_{U_{k}}||T})$ as all the private keys of the user $U_{k}$ in T periods, the user $U_{k}$ generates all the private keys and stores the private key set ${SK}_{U_{k}}$. Then calculate ${Ā}_{{ID}_{U_{k}}||i}=A_{{ID}_{U_{k}}||i}+{A’}_{{ID}_{U_{k}}||i}$, $T_{{ID}_{U_{k}}||i}={Ā}_{{ID}_{U_{k}}||i}\cdot {SK}_{{ID}_{U_{k}}||i}$, then the public key of the user $U_{k}$ in the i-th period is ${PK}_{{ID}_{U_{k}}||i}=({Ā}_{{ID}_{U_{k}}||i},T_{{ID}_{U_{k}}||i})$. The public key set of the user $U_{k}$ in the T-period is ${PK}_{U_{k}}=({PK}_{{ID}_{U_{k}}||0},{PK}_{{ID}_{U_{k}}||1},\cdots \cdots {,PK}_{{ID}_{U_{k}}||T})$. After the user $U_{k}$ generates the public key set, he stores ${PK}_{U_{k}}$ carefully at first, and then publishes the public key together with the signature after signing.

#### 3.1.4. Key Update

The user $U_{k}$ updates the key, Update(q,n) → (${SK’}_{U_{k}},{PK’}_{U_{k}}$): To ensure the security of the signature system, users are advised to update their keys periodically. Under the circumstances in which the user key is not leaked and is still within the T-period, the user continues to use the original master key without PKG updating. To generate a new user master key in such cases, only step 3 in Section 3.1.1 needs to be repeated, followed by the calculation of key iteration as described in Section 3.1.2. When the key is used up or the key is leaked, the user sends a key request to PKG again to update the master key, i.e., the user will redo all the steps in Section 3.1.1 and Section 3.1.2 to update the key. Since the lattice-basis delegation algorithm takes less time to calculate than the trapdoor-generation algorithm, it will complete the calculation task quickly, which ensures that the user can update the key in a relatively short time.

### 3.2. Strong Forward-Secure Signature Scheme on Lattice

This section provides a detailed description of a strong forward-secure signature scheme. The construction of the signature scheme is based on the strong forward-secure key-iteration algorithm KI put forward in Section 3.1. It guarantees strong forward security of signatures under a quantum attack environment.

#### 3.2.1. Parameter Generation

The strong forward-secure signature scheme on the lattice is composed of two entities, the identity-based cryptosystem IBC user and the key generation center PKG. When the user needs to obtain the key, he sends a key request to PKG, which includes the user’s ID and the period T of the required key. PKG will execute the parameter generation algorithm as soon as it receives the key request:

Setup(n) → PP: PKG inputs the security parameter n, and randomly selects the prime number q, m>64+nlogn/log3, three sets of Gaussian parameters $\sigma =(\sigma_{0},\sigma_{1},\cdots \cdots ,\sigma_{T})$, $\sigma ’=({\sigma ’}_{0},{\sigma ’}_{1},\cdots \cdots ,{\sigma ’}_{T})$,$\delta =(\delta_{0},\delta_{1},\cdots \cdots ,\delta_{T})$and a hash function $H_{2}:{\left\{0,1\right\}}^{*}\to \left\{v:v\in {\left\{-1,0,1\right\}}^{k},||v||\leq k\right\}$. After that PKG publishes the parameters $PP=(n,q,m,\sigma ,{\sigma ’,\delta ,H}_{2})$.

#### 3.2.2. Key Generation

Suppose the user is $U_{k}$, the user’s identity ID is ${ID}_{U_{k}}$, and the required key period is T. The user invokes the strong forward-secure key-iteration algorithm in 3.1 to generate a signature key:

KeyGen(PP) → (${SK}_{U_{k}}$,${PK}_{U_{k}}$): Inputting the security parameter PP, the user invokes the key-iteration algorithm in Section 3.1.2 to generate a private key set and a public key set (${SK}_{U_{k}}$,${PK}_{U_{k}}$) for T periods. The user $U_{k}$ first stores the set of public keys, and subsequently publishes the public key ${PK}_{U_{k}}$ of the current period along with its signature after signing. After the T-period public–private key set is used up, the user $U_{k}$ invokes the key update algorithm in Section 3.1.3 to generate another T’-period public–private key set (${SK’}_{U_{k}},{PK’}_{U_{k}}$) for a new round of signature and verification.

#### 3.2.3. Sign

When a user intends to sign a message, he checks the private key number in the private key set to determine the current period. He then publicizes the public key of the period along with the signature. The private key will become invalid once being used, because the user will delete the used private key from the private key set. This allows the period to be determined from the label of the private key.

Sign(PP,m,${SK}_{{ID}_{U_{k}}||i}$) → $e_{i}$: Assuming that the current period is i and the user is $U_{k}$, then $U_{k}$ uses the private key of the i-th period ${SK}_{{ID}_{U_{k}}||i}$ to sign the message m. The user $U_{k}$ signature needs to do the following work:

1. The user inputs the public parameters PP, the message $m\in {\left\{0,1\right\}}^{*}$, and the private key of the i-th period ${SK}_{{ID}_{U_{k}}||i}$.
2. The user randomly selects a vector $y_{i}\leftarrow D_{r_{i}}^{m}$.
3. Calculates $c_{i}=H_{2}({Ā}_{{ID}_{U_{k}}||i}\cdot y_{i},m)$.
4. Then calculates $z_{i}={SK}_{{ID}_{U_{k}}||i}\cdot c_{i}+y_{i}$.
5. Outputs the current period signature $e_{i}=(c_{i}{,z}_{i})$ with a probability of $min(1,\frac{D_{r_{i}}^{m}(z_{i})}{{MD}_{r_{i,{SK}_{ID||i}\cdot c_{i}(z_{i})}}^{m}})$, and re-executes the algorithm if there is no output.
6. Publishes the current period public key ${PK}_{{ID}_{U_{k}}||i}$.

#### 3.2.4. Verify

The user $U_{k}$ signs the message, the verifier needs to verify the signature to confirm the validity of the signature.

Verify(PP,m,$e_{i},{PK}_{{ID}_{U_{k}}||i}$) → 0/1:The verifier inputs the public parameter PP, the original message m, the public key ${PK}_{{ID}_{U_{k}}||i}$ disclosed by user $U_{k}$ and the signature $e_{i}$, then the verifier performs the following operations:

If $c_{i}=H_{2}({Ā}_{{ID}_{U_{k}}||i}\cdot z_{i}-T_{{ID}_{U_{k}}||i}\cdot c_{i},m)$ and the $z_{i}\leq 2r_{i}\cdot \sqrt{m}$ are established simultaneously, the signature is accepted and the output result is 1, otherwise, the signature is rejected and the output result is 0.

Theorem 1 will help to prove the correctness of the identity-based strong forward-secure signature scheme brought forward in this paper.

**Theorem** **1.** *The verification process of the signature guarantees the correctness of the signature.*

**Proof** **of** **Theorem 1.** The public key is ${PK}_{{ID}_{U_{k}}||i}=({Ā}_{{ID}_{U_{k}}||i},T_{{ID}_{U_{k}}||i})$, the signature is $e_{i}=(c_{i},z_{i})$, the message is m, and the public key and message signature pair are public. The correctness of the verifier’s success in verifying the signature is guaranteed by the following equation:

$$\begin{array}{l} H_{2}({Ā}_{{ID}_{U_{k}}||i}\cdot z_{i}-T_{{ID}_{U_{k}}||i}\cdot c_{i},m)\\ =H_{2}({Ā}_{{ID}_{U_{k}}||i}\cdot ({SK}_{{ID}_{U_{k}}||i}\cdot c_{i}+y_{i})-{Ā}_{{ID}_{U_{k}}||i}\cdot {SK}_{{ID}_{U_{k}}||i}\cdot c_{i},m)\\ =H_{2}({Ā}_{{ID}_{U_{k}}||i}\cdot y_{i},m)\\ =c_{i} \end{array}$$

□

By verifying the signature, it confirms that the signature is indeed generated by the holder of the private key, which guarantees both data integrity and unaltered transmission, therefore ensuring the accuracy of the signature.

## 4. Performance Analysis

### 4.1. Existential Unforgeability against Chosen-Message Attacks

Theorem 2 will help to prove the existential and unforgeability of the identity-based strong forward-secure signature scheme proposed in this paper.

Under the hard assumption of the SIS problem on the lattice, it is proved that the identity-based strong forward-secure signature scheme on the lattice is existentially unforgeable.

**Theorem** **2.** *Under the random oracle model, according to the difficulty assumption of the SIS problem, the identity-based strong forward-secure signature scheme on the lattice realizes the existential unforgeability under the chosen-message attacks.*

**Proof of** **Theorem 2.** Assume that there is an adversary A of PPT who outputs a forged signature with a non-negligible probability after a polynomial query, which destroys the unforgeability of the identity-based strong forward-secure signature scheme given in 3.2. Then a simulator C with non-negligible advantages will be constructed, which can solve the SIS problem instance. □

Parameter establishment: C selects two hash functions $H_{1}:{\left\{0,1\right\}}^{*}\to Z^{m\times m}$, $H_{2}:{\left\{0,1\right\}}^{*}\to \left\{v:v\in {\left\{-1,0,1\right\}}^{k},||v||\leq k\right\}$, and generates matrices $M_{U_{k}A_{0}},M_{U_{k}B_{0}}\in Z_{q}^{n\times m}$ and $M_{U_{k}T_{A_{0}}},M_{U_{k}T_{B_{0}}},U_{kT_{A_{0}}},U_{kT_{B_{0}}}\in Z^{m\times m}$, then sends $\left(M_{U_{k}A_{0}},M_{U_{k}B_{0}},H_{1},H_{2}\right)$ to A.

$H_{1}$ Query: For any time period i(i=1,2,…,T), the simulator C maintains two list $L_{1}=({ID}_{U_{k}}||U_{kT_{A_{0}}}||i,Q_{i})$, ${L’}_{1}=({ID}_{U_{k}}||U_{kT_{B_{0}}}||i,O_{i})$ of $H_{1}$ query, where $Q_{i}$ represented the hash value of ${ID}_{U_{k}}||U_{kT_{A_{0}}}||i$, $O_{i}$ represented he hash value of ${ID}_{U_{k}}||U_{kT_{B_{0}}}||i$, in which the initial lists are empty. A will conduct $H_{1}$ query on $\left({ID}_{U_{k}}||U_{kT_{A_{0}}}||i,Q_{i}\right)$, if the tuple $\left({ID}_{U_{k}}||U_{kT_{A_{0}}}||i,Q_{i}\right)$ is in $L_{1}$, C will use $Q_{i}$ as the response to the $H_{1}$ query, otherwise C will randomly choose a $G_{i}\in Z_{q}^{m\times m}$ and use $G_{i}$ as the response to the $H_{1}$ query, after that $\left({ID}_{U_{k}}||U_{kT_{A_{0}}}||i,G_{i}\right)$ will be added into $L_{1}$. A will conduct $H_{1}$ query on $\left({ID}_{U_{k}}||U_{kT_{B_{0}}}||i,O_{i}\right)$ , if $\left({ID}_{U_{k}}||U_{kT_{B_{0}}}||i,O_{i}\right)$ is in ${L’}_{1}$, C will use $O_{i}$ as the response to the $H_{1}$ query, otherwise C will randomly choose a $J_{i}\in Z_{q}^{m\times m}$ and use $J_{i}$ be the response to the $H_{1}$ query, whereupon $\left({ID}_{U_{k}}||U_{kT_{B_{0}}}||i,J_{i}\right)$ will be added into ${L’}_{1}$.

$H_{2}$ Query: C maintains a list $L_{2}=({Ā}_{{ID}_{U_{k}}||i}\cdot y_{i},c_{i}$) of $H_{2}$ query, and the initial list is empty. A will conduct $H_{1}$ query on $({Ā}_{{ID}_{U_{k}}||i}\cdot y_{i},c_{i}$), if $({Ā}_{{ID}_{U_{k}}||i}\cdot y_{i},c_{i}$) is in $L_{2}$, C will respond $c_{i}$ as the response of $H_{2}$ query, otherwise C will randomly choose a $C_{i}\in Z_{q}^{k}$ and use it as the response to the $H_{2}$ query, and then (${Ā}_{{ID}_{U_{k}}||i}\cdot y_{i},C_{i}$) will be added into $L_{2}$.

Key query: C maintains a list $L_{3}=({ID}_{U_{k}}||U_{kT_{A_{0}}}||i,{ID}_{U_{k}}||U_{kT_{B_{0}}}||i,{Ā}_{{ID}_{U_{k}}||i},{SK}_{{ID}_{U_{k}}||i})$, and the initial list is empty. C responds to the initial or iterative key query as follows:

1. C first browses whether there is a corresponding hash value in the list $L_{1}$ and ${L’}_{1}$, if exists, directly returns the corresponding hash value and calculates $A_{{ID}_{U_{k}}||i}=M_{U_{k}A_{0}}\cdot {\left(H_{1}({ID}_{U_{k}}||U_{kT_{A_{0}}}||0)H_{1}({ID}_{U_{k}}||U_{kT_{A_{0}}}||1){\cdots \cdots H}_{1}({ID}_{U_{k}}||U_{kT_{A_{0}}}||i)\right)}^{-1}$, ${A’}_{{ID}_{U_{k}}||i}=M_{U_{k}B_{0}}\cdot {\left(H_{1}({ID}_{U_{k}}||U_{kT_{B_{0}}}||T)H_{1}({ID}_{U_{k}}||U_{kT_{B_{0}}}||T-1{)\cdots \cdots H}_{1}({ID}_{U_{k}}||U_{kT_{B_{0}}}||i)\right)}^{-1}$. If the corresponding hash value does not exist, C randomly select a matrix $P_{i}\in Z_{q}^{n\times m}$, then run the BasisDel algorithm to generate a private key ${SK}_{{ID}_{U_{k}}||i}$and add it to the list $L_{3}$.
2. C maintains list $L_{4}=({ID}_{U_{k}}||U_{kT_{A_{0}}}||i,{ID}_{U_{k}}||U_{kT_{B_{0}}}||i,{SK}_{{ID}_{U_{k}}||i}{,sk}_{{ID}_{U_{k}}||i-1}{,sk}_{{ID}_{U_{k}}||i+1}$), if A performs a key query on ${ID}_{U_{k}}||$i, C returns the current cycle private key ${SK}_{{ID}_{U_{k}}||i}$ of A as a response. Then C browses whether there is a corresponding hash value in the list $L_{1}$ and ${L’}_{1}$, and if so, directly returns the corresponding hash value. After that calculate $R_{i+1}=H_{1}({ID}_{U_{k}}||U_{kT_{A_{0}}}||i+1)$, $R_{i-1}=H_{1}({ID}_{U_{k}}||U_{kT_{A_{0}}}||i-1)$, ${R’}_{i+1}=H_{1}({ID}_{U_{k}}||U_{kT_{B_{0}}}||i+1)$, ${R’}_{i-1}=H_{1}({ID}_{U_{k}}||U_{kT_{B_{0}}}||i-1)$. If the corresponding hash value does not exist, C randomly selects a matrix $G_{i}\in Z_{q}^{m\times m}$, then runs the BasisDel algorithm to generate a forward private key ${sk}_{{ID}_{U_{k}}||i-1}$ and a backward private key ${sk}_{{ID}_{U_{k}}||i+1}$, afterwards adds them into list $L_{4}$.

Signature query: Adversary A asks for the signature of message m, B first browses the list $L_{1}$, ${L’}_{1}$ and $L_{2}$, for any period i ≤ T, if there is a corresponding hash value, then C calculates $z_{i}={SK}_{{ID}_{U_{k}}||i}\cdot c_{i}+y_{i}$ and outputs the current period signature $e_{i}=(c_{i},z_{i})$ with the probability of $min(1,\frac{D_{r_{i}}^{m}(z_{i})}{{MD}_{r_{i,{SK}_{ID||i}\cdot c_{i}(z_{i})}}^{m}})$; otherwise, C randomly selects the vector ${c’}_{i}$ and ${z’}_{i}$, whereupon obtained $c_{i}$ by $H_{2}$ query with $H_{2}({Ā}_{{ID}_{U_{k}}||i}\cdot {z’}_{i}-T_{{ID}_{U_{k}}|i}\cdot {c’}_{i},m)$, and then computed $z_{i}={SK}_{{ID}_{U_{k}}||i}\cdot c_{i}+y_{i}$ to output the current period signature $e_{i}=(c_{i},z_{i})$.

Forgery: The adversary ends the above queries, outputs the identity ${ID}_{U_{k}}^{*}$ of current period $i^{*}$, message $m^{*}$ and signature of the current period $e_{i^{*}}$. The adversary wins if the following conditions hold.

1. 1 $\leq i^{*}\leq$ T.
2. ${ID}_{U_{k}}^{*}$ has not been queried in the key query.
3. (${ID}_{U_{k}}^{*}$,$i^{*}{,m}^{*}$) has not been asked in the signature query.
4. Signature $e_{i^{*}}$ pass the verification.

According to the Fork Lemma in the security proof, when adversary A successfully forges a signature $e_{i^{*}}$ and is used by simulator C to crack a difficult problem, the challenge process needs to be run twice so that the output of both processes matches for a period of time before diverging at a certain point. This allows simulator C to solve the difficult problem. So there exists the following equation ${Ā}_{{ID}_{U_{k}}||i}\cdot z_{i}-T_{{ID}_{U_{k}}||i}\cdot c_{i}=Ā\cdot z_{i^{*}}-T_{{{ID}_{U_{k}}}^{*}||i^{*}}\cdot c_{i^{*}}$, where $T_{{ID}_{U_{k}}||i}={Ā}_{{ID}_{U_{k}}||i}\cdot {SK}_{{ID}_{U_{k}}||i}$, $T_{{{ID}_{U_{k}}}^{*}||i^{*}}=Ā\cdot {SK}_{{{ID}_{U_{k}}}^{*}||i^{*}}$. Transform the equation to obtain (${Ā}_{{ID}_{U_{k}}||i}\cdot z_{i}-{Ā}_{{ID}_{U_{k}}||i}\cdot {SK}_{{ID}_{U_{k}}||i}\cdot c_{i})-({Ā}_{{ID}_{U_{k}}^{*}||i^{*}}\cdot z_{i^{*}}-{Ā}_{{ID}_{U_{k}}^{*}||i^{*}}\cdot {SK}_{{ID}_{U_{k}}^{*}||i^{*}}\cdot c_{i^{*}})$ = 0, because of the collision resistance of the hash function, there obtains $A_{0}({\left(R_{{ID}_{U_{k}}||i}\right)}^{-1}\cdot y_{i}-{\left(R_{{ID}_{U_{k}}^{*}||i^{*}}\right)}^{-1}\cdot y_{i^{*}})=0$, $B_{0}({\left({R’}_{{ID}_{U_{k}}||i}\right)}^{-1}\cdot y_{i}-{\left({R’}_{{ID}_{U_{k}}^{*}||i^{*}}\right)}^{-1}\cdot y_{i^{*}})=0$. Let $\lambda_{1}=({\left(R_{{ID}_{U_{k}}||i}\right)}^{-1}\cdot y_{i}-{\left(R_{{ID}^{*}||i^{*}}\right)}^{-1}\cdot y_{i^{*}}$), $\lambda_{2}=({\left({R’}_{{ID}_{U_{k}}||i}\right)}^{-1}\cdot y_{i}-{\left({R’}_{{ID}_{U_{k}}^{*}||i^{*}}\right)}^{-1}\cdot y_{i^{*}}$), $\lambda_{1}$ and $\lambda_{2}$ are both non-zero vectors, and there are $A_{0}\lambda_{1}$ = 0 and $B_{0}\lambda_{2}$ = 0, so $\lambda_{1}$ and $\lambda_{2}$ will be regarded as the solution to the SIS problem.

If there exists an adversary that can forge a valid signature of a digital signature scheme with probability acc, then there exists an algorithm $F_{B}$ that outputs the solution of the SIS problem instance with probability Adv ≥ acc$\cdot (\frac{acc}{q_{H_{1}}+q_{H_{2}}}-\frac{1}{h})$ by exploiting the capacity of the adversary, where acc ≥ε-$\frac{q_{s}({q_{H_{1}}+q_{H_{2}+}q}_{s}+1)}{2^{k}}$, $q_{H_{1}}$ and $q_{H_{2}}$, respectively, represent the number of $H_{1}$ and $H_{2}$ query, $q_{s}$ represent the number of signature queries, h is the number of replies to random oracle queries. In this way, the simulator cracks the SIS problem with a non-negligible advantage, but because of the computational difficulty of the SIS problem, such an adversary cannot break through our scheme, so the scheme is secure.

### 4.2. Strong Forward Security

#### 4.2.1. Forward-Security Analysis

1. Key-iteration algorithm has forward security

The user’s signature private key iterates as the period increases. If an attacker obtains the user $U_{k}$’s signature private key ${SK}_{{ID}_{U_{k}}||j}$ of period j and wants to use the signature private key ${SK}_{{ID}_{U_{k}}||j}$ to obtain the private key ${SK}_{{ID}_{U_{k}}||j-1}$ of period j − 1, then the attacker needs to break through the problem of small integers on the lattice. As the computational difficulty of the problem, the attacker cannot obtain the private key ${SK}_{{ID}_{U_{k}}||j-1}$ used by ${SK}_{{ID}_{U_{k}}||j}$, as well as being unable to obtain the private keys such as ${SK}_{{ID}_{U_{k}}||j-2}$, ${SK}_{{ID}_{U_{k}}||j-3}$,…, ${SK}_{{ID}_{U_{k}}||1}$ for the existing signatures.

2. The signature scheme is forward-secure

The user $U_{k}$’s signature in the j-th period is $e_{j}=(c_{j}{,z}_{j})$, where $c_{j}=H_{2}({Ā}_{{ID}_{U_{k}}||i}\cdot y_{i},m)$, $z_{j}={SK}_{{ID}_{U_{k}}||j}\cdot c_{j}+y_{j}$, m is the message, ${PK}_{{ID}_{U_{k}}||j}=({Ā}_{{ID}_{U_{k}}||j},T_{{ID}_{U_{k}}||j})$ is the public key, and $y_{j}$ is selected randomly. The attacker wants to forge the signature of period j − 1. Since the public key is public, the attacker has the condition to calculate $c_{j-1}$. If he wants to forge the signature, the attacker needs to calculate it $z_{j-1}$. At this time, the private key of period j − 1 is needed. Due to the difficulty of solving the problem with small integers on the grid, even if the attacker obtains the signature key of period j, he cannot forge the signature key of period j − 1, so a valid signature cannot be generated. The statements mentioned above ensure the forward security of the signature.

#### 4.2.2. Backward Security Analysis

1. The key-iteration algorithm has backward security

The user’s signature private key iterates as the period decreases. If an attacker obtains the user $U_{k}$’s signature private key ${SK}_{{ID}_{U_{k}}||j}$ of period j and wants to use the signature private key ${SK}_{{ID}_{U_{k}}||j}$ to obtain the private key ${SK}_{{ID}_{U_{k}}||j+1}$ of period j + 1, then it needs the attacker to break through the small integer problem on the lattice, so the attacker cannot obtain the private key ${SK}_{{ID}_{U_{k}}||j+1}$ using ${SK}_{{ID}_{U_{k}}||j}$, as well as being unable to obtain the private keys such as ${SK}_{{ID}_{U_{k}}||j+2}$,${SK}_{{ID}_{U_{k}}||j+3}$,…,${SK}_{{ID}_{U_{k}}||T}$ for the subsequent signatures.

2. The signature scheme is forward-secure

The user $U_{k}$’s signature in the j-th period is $e_{j}=(c_{j}{,z}_{j})$, where $c_{j}=H_{2}({Ā}_{{ID}_{U_{k}}||i}\cdot y_{i},m)$, $z_{j}={SK}_{{ID}_{U_{k}}||j}\cdot c_{j}+y_{j}$, m is the message, ${PK}_{{ID}_{U_{k}}||j}=({Ā}_{{ID}_{U_{k}}||j},T_{{ID}_{U_{k}}||j})$ is the public key, and $y_{j}$ is selected randomly. The attacker wants to forge the signature of period j+1. If the user $U_{k}$ has signed with ${SK}_{{ID}_{U_{k}}||j+1}$, the public key has been disclosed by the user $U_{k}$, then the attacker has the condition to calculate $c_{j+1}$. If he wants to forge the signature, the attacker still needs to calculate $z_{j+1}$. At this time, the private key of period j + 1 is needed. Due to the difficulty of solving the problem with small integers on the grid, even if the attacker obtains the signature key of period j, he cannot forge the signature key of period j + 1, so a valid signature cannot be generated. This ensures the forward security of the signature. If the user $U_{k}$ has not used ${SK}_{{ID}_{U_{k}}||j+1}$ to sign, then the user $U_{k}$ has not disclosed the public key. With the anti-collision property of the hash function, the attacker cannot calculate $c_{j}$, let alone calculate $z_{j}$. Therefore, a valid signature cannot be generated, thus ensuring the forward security of the signature.

Since the key-iteration algorithm and the signature scheme have both forward security and backward security, it is shown that the scheme proposed in Section 3 has strong forward security.

## 5. Remote Identity Authentication to Resist Quantum Attacks

With the popularity of the Internet, it has become more convenient and effective to use the Internet to engage in various activities, which inevitably requires the credibility of the participants. To ensure consistency between the users’ real identity and the digital identity on the network, it is necessary to use some technical verification methods for consistency verification. Identity-authentication technology solves the problem of consistency. It is an effective means to ensure information security. It plays an important role in information systems and is used as a tool to confirm the validity of participants’ identities.

### 5.1. Overview of Remote Identity Authentication

Remote identity authentication is the process of verifying a user’s identity through a network or remote communication channel. It allows users to authenticate without having to attend in person to gain access to systems or resources. Remote identity authentication includes static authentication, dynamic authentication, and multi-factor authentication [29]. It has been practiced in some public domains and has become a common authentication method. The dynamic password authentication of digital signatures plays a significant part in many fields because of its particularity and real-time characteristics [30].

Applying the digital signature scheme to the working process of remote identity dynamic authentication could guarantee the security of the authentication process. The whole process includes two stages of registration and authentication. In the registration stage, the user stores his information on the server. In the authentication stage, the user and the server interact to prove their identity [31]. This section applies the identity-based strong forward-secure signature scheme proposed in Section 3 to the remote identity-authentication process to implement a secure remote identity-authentication scheme.

The lattice-based signature scheme utilizes mathematical problems based on lattices as the fundamental security measure. The signature scheme proposed in Section 3 is specifically built upon the SIS problem, which poses a formidable challenge that currently remains unsolved by quantum computers. Therefore, the lattice-based signature scheme has strong security under quantum computer attacks.

### 5.2. Lattice-Based Strong Forward-Secure Signature Scheme for Remote Authentication

In the remote identity-authentication process based on the signature scheme, if only a pair of public and private keys are generated when the user registers, then the signature private key used by the user in each authentication process will remain unchanged. If the key is leaked, the entire authentication process will no longer be safe. At this time, the user signature system needs to be updated, otherwise a malicious third party may obtain the important information of the user stored on the server.

**Figure 2:**
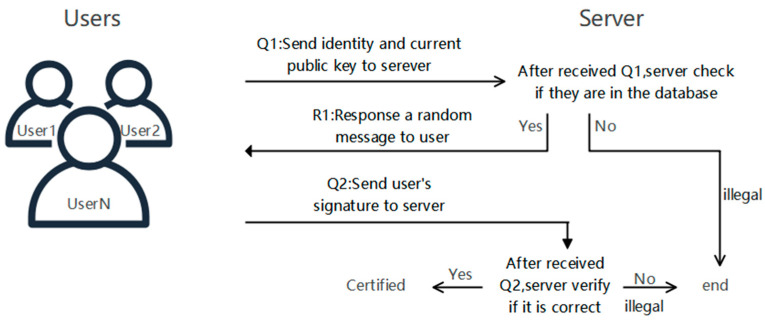
Remote identity-authentication framework.

However, it will be inconvenient to update the user signature system. Applying an identity-based strong forward-secure signature scheme to remote user authentication can reduce the impact of key leakage. In the identity-based strong forward-security signature scheme, there will be a unique key pair for signing and verification in each period, so even if a private key is accidentally leaked due to user storage, it ensures that the user’s subsequent identity authentication is safe. With the strong forward security of the signature private key, the attacker cannot calculate the private key of other periods through a certain private key, so he cannot pretend to be a legitimate user for authentication. The remote identity-authentication framework of lattice-based strong forward-secure signature scheme is shown in Figure 2.

#### 5.2.1. Enrollment Phase

When the user registers, first, they are supposed to send the identity information to PKG to obtain the master private key and master public key, and then the user’s master private key and master public key generate a public–private key set. After that, the user sends his identity and public key set to the server. When receiving the encrypted information, the server uses the private key to decrypt to obtain the user identity and public key set, and then stores it in the server database. The specific registration process is:

1. The user $U_{k}$ first determines the required period T, initiates a key request to PKG to obtain the master private key $M_{U_{k}T_{A_{0}}}$ and the master public key $M_{U_{k}T_{B_{0}}}$, and then the user $U_{k}$ uses $M_{U_{k}T_{A_{0}}}$, $M_{U_{k}T_{B_{0}}}$ to generate the private key set ${SK}_{U_{k}}$ and the public key set ${PK}_{U_{k}}$, after that stores the private key set and the public key set carefully.
2. The server uses a public-key encryption algorithm to generate a public–private key pair (ssk, spk), and sends the public key to the user $U_{k}$ to encrypt the transmitted identity information.
3. The user $U_{k}$ uses the public key of the server to encrypt the identity ${ID}_{U_{k}}$ and the public key set ${PK}_{U_{k}}$ with spk and then sends them to the server.
4. The server uses the ssk to decrypt and obtains the user’s sum ${ID}_{U_{k}}$ and store ${PK}_{U_{k}}$ it in the server’s database.

After the registration is completed, the user $U_{k}$ becomes a legal user of the server and performs remote identity authentication through the server.

#### 5.2.2. Authentication Phase

**Figure 3:**
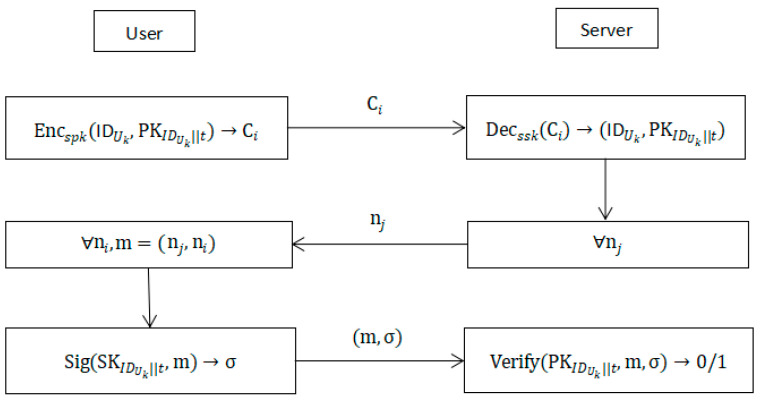
Remote authentication of lattice-based strong forward-secure signature scheme.

The user $U_{k}$ proves his identity with the server through the following interactions:

1. The user $U_{k}$ checks the private key number in the private key set to determine the current period t(t≤T), encrypts the user identity ${ID}_{U_{k}}$ as well as the public key corresponding to the current period ${PK}_{{ID}_{U_{k}}||t}$ with the server’s public key spk, and sends it to the server.
2. After receiving the ciphertext sent by ${ID}_{U_{k}}$ the user, the server decrypts it with the private key ssk to obtain the user’s $U_{k}$ and the public key of the current period ${PK}_{{ID}_{U_{k}}||t}$, and then the server compares the user’s identity and public key in the database to see whether they are consistent with the stored ones. If they are consistent, continue 3, otherwise stop the interaction.
3. The server randomly selects a challenge message and sends the challenge message to the user.
4. The user replies to the challenge information, and takes the challenge information and replies to information as messages to be signed.
5. Use the private key of the current period ${SK}_{{ID}_{U_{k}}||t}$ to sign, and send the message signature pair to the server after signing.
6. After the server receives the message signature pair, the public key ${PK}_{{ID}_{U_{k}}||t}$ is used to verify. If the signature is verified, the user is authenticated; otherwise, the authentication fails. The remote identity-authentication process of the lattice-based strong forward-secure signature scheme is shown in Figure 3.

## 6. Conclusions

In a digital signature scheme, if the key is leaked, the signature scheme will be insecure. To reduce the impact of leaked keys on the security of a signature scheme, a strong forward-secure signature scheme is proposed in this paper. With the emergence of quantum computing, the security of schemes based on RSA and discrete logarithm problems is corrupt. Therefore, a strong forward-secure signature scheme that is resistant to quantum attacks is proposed in this paper. The trapdoor-generation algorithm, lattice-basis delegation technology, and hash function are used to distribute a unique key pair for every period by iterating the key. The above algorithms ensure the forward security and backward security of the key, so that the key has strong forward security. Under the random oracle model, the proposed signature scheme satisfies existential unforgeability based on the difficulty assumption of the SIS problem. This paper is about a lattice-based strong forward-secure signature scheme under the random oracle model. In the future, a lattice-based strong forward-secure signature scheme under the standard model will be further studied.

## Figures and Tables

**Table 1:** Symbols in Strong Forward-Secure Signature Scheme.

Symbol	Meaning
${ID}_{U_{k}}$	The identity of user K
$M_{U_{k}T_{A_{0}}}$	User K’s master private key
$M_{U_{k}T_{B_{0}}}$	User K’s master public key
${sk}_{{ID}_{U_{k}}||0}$	User K’s initial forward private key
${sk’}_{{ID}_{U_{k}}||0}$	User K’s initial backward private key
${SK}_{{ID}_{U_{k}}||t}$	The private key of user K in period t
${PK}_{{ID}_{U_{k}}||t}$	The public key of user K in period t
PKG	key generation center
$e_{i}$	signature

## Data Availability

Not applicable.
